# Pediatric perioperative hypersensitivity in Denmark: A 20 year retrospective analysis

**DOI:** 10.1111/pai.70079

**Published:** 2025-03-31

**Authors:** Cecilie N. Madsen, Birgitte Bech Melchiors, Holger Mosbech, Kirsten Skamstrup Hansen, Lene H. Garvey

**Affiliations:** ^1^ Danish Anaesthesia Allergy Centre, Allergy Clinic, Department of Dermatology and Allergy Copenhagen University Hospital‐Herlev and Gentofte Copenhagen Denmark; ^2^ Department of Pediatrics Herlev and Gentofte Hospital, University of Copenhagen Herlev Denmark; ^3^ Department of Clinical Medicine University of Copenhagen Copenhagen Denmark

**Keywords:** allergy, anaphylaxis, anesthesia, children, drug allergy, pediatric, perioperative anaphylaxis, perioperative hypersensitivity, risk stratification

## Abstract

**Background:**

Perioperative hypersensitivity (POH) is rare but potentially life‐threatening, and data on POH in children are sparse. This single‐center study aimed to describe clinical presentations and allergy investigations in children with suspected POH in Denmark, and to evaluate a risk stratification algorithm used for suspected POH (elevated tryptase, 2 or more organ systems involved and urticaria/angioedema).

**Methods:**

Retrospective data from 70 children who had undergone allergy investigations at the specialized Danish Anaesthesia Allergy Centre were included. Children were divided into a test positive and test negative group based on results from allergy investigations, and the groups were compared. Sensitivities, specificities, positive and negative predictive values were calculated for the risk stratification algorithm for suspected POH and for different combinations of symptoms.

**Results:**

24% of the children tested positive. The most confirmed allergen was chlorhexidine (*n* = 3), followed by NMBAs (*n* = 2) and antibiotics (*n* = 2). Skin symptoms were most common (94%), and cardiovascular symptoms (CVS) appeared as the first symptom in 50% of the test positive children. CVS were more common in the test positive group. The risk stratification algorithm had a high sensitivity (88%) but a higher sensitivity (94%) was seen in the combination “elevated tryptase or CVS or urticaria/angioedema”.

**Conclusion:**

Several different allergens were confirmed in this study, including hidden allergens like chlorhexidine. This emphasizes the need to identify all potential allergens and the need for investigation at a specialized allergy center. More data are needed to make recommendations on the optimal risk stratification algorithm in children with suspected POH.

**Key message**

Perioperative hypersensitivity in children is rare but reactions can be severe with hypotension as the first symptom. Anesthesiologists should be aware of anaphylaxis as a differential diagnosis to perioperative hypotension and refer children for allergy investigations when perioperative hypersensitivity is suspected.

## 1 INTRODUCTION

Perioperative hypersensitivity (POH) is rare, with pediatric cases being even more uncommon. Reported incidences vary depending on geography and regional definitions of POH.^1^, ^2^ Severe perioperative events are estimated in a large multicenter European study to occur in 5.2% of pediatric surgeries, with anaphylaxis occurring in 1 per 10,000 pediatric cases.^3^ In the data from the National Audit Project 6 (NAP6) study, serious perioperative allergic reactions had a reported incidence of 2.7 per 100,000 pediatric surgeries.^4^

Symptoms of POH can resemble other adverse events during anesthesia.^1^, ^5^ Early recognition and correct treatment, as well as referral for allergy assessment, are essential for correct diagnosis and for securing the safety of subsequent surgery. Due to the complexity of a suspected allergic reaction in the perioperative setting, requiring a detailed account of exposures and sequence of events and knowledge about subsequent allergy investigations, allergy work‐up should ideally be carried out in specialized centers with an established collaboration between allergologists and anesthesiologists.^1^

Most published studies on POH are based on adult populations, and only a few recent reviews address the pediatric population.^6^, ^7^, ^8^ Data on pediatric POH consists mostly of case series, supplemented by cohort studies from different countries.^5^, ^9^, ^10^, ^11^, ^12^, ^13^ However, due to the rarity of POH in children, cohorts are generally small, based on multicenter studies, and data may lack consistency in testing or symptom reporting. Additionally, many studies only present data from severe reactions.

A risk stratification algorithm for identifying POH has been proposed in 2020.^14^ It includes three criteria: elevated tryptase, two or more organ systems involved, and urticaria/angioedema. If a patient meets at least one of these criteria, they are considered more likely to have POH. This risk stratification algorithm was evaluated in 2022^15^ in a mixed adult and pediatric population, showing a sensitivity of 98.8% and a specificity of 34.6%. However, the risk stratification algorithm has not been tested in an exclusively pediatric population.

This study presents data from a Danish single‐center pediatric population with suspected POH and aims to (1) describe demographics and clinical characteristics, and (2) present the outcomes of allergy investigation. Furthermore, this study seeks to evaluate the risk stratification algorithm used to investigate suspected perioperative allergic reactions.^14^

## 2 METHODS

### 2.1 Population and data collection

In this retrospective single center study, children (<18 years at time of reaction) investigated for suspected perioperative allergic reactions at the Danish Anaesthesia Allergy Centre (DAAC) from 2004 to 2023 were included. DAAC is the national reference center for POH in Denmark, and data on suspected allergic reactions and results of subsequent allergy investigations are stored in a database. Out of the 843 patients investigated in DAAC over the 20‐year period, 70 (8.3%) were pediatric patients, and they were included in this study. If database records were not complete, supplementary data were retrieved from patient records. Patients were divided into a test positive and a test negative group, depending on the outcome of the allergy investigation.

The database is approved by the Capital Region of Denmark with journal number P‐2022‐317. Approval for accessing patient journals with exemption from obtaining patient consent was granted with journal number R‐23022410.

### 2.2 Allergy investigation

In DAAC, referred patients undergo systematic investigations including skin tests, in vitro tests, and drug provocation tests. Evaluations are performed by a combination of allergologists and anesthesiologists and include the collection of their anesthesia records and all available information about the observed reaction to aid the identification of potential culprit allergens. Tryptase levels during the reaction are compared to baseline tryptase to assess mast cell activation. The formula for elevated tryptase used in this study is the internationally recommended algorithm: acute serum tryptase >1.2*basal serum tryptase+2.^16^ In this study, elevated reaction tryptase levels were included in analyses regardless of timing, but reaction tryptase was excluded in five patients as it was not elevated and taken more than 2 h after the suspected allergic reaction. The two‐hour cut‐off is based on recommendations in a recent review on the use of tryptase in perioperative hypersensitivity.^17^

On referral of patients to DAAC, the American Society of Anesthesiologists classification (ASA) is recorded. Reaction severity is graded using the Ring and Messmer scale^16^ based on the severity of the reaction. An individual plan for testing is made based on medications administered before the reaction, including disinfectants and latex, regardless of whether exposure to these was documented. Testing includes blood samples for specific IgE for drugs where it is available. Skin prick tests (SPT) are performed with all potential allergens, and if the child can cooperate, intradermal tests (IDT) are titrated up to published non‐irritant concentrations.^16^

In general, a full allergy workup includes in vitro tests supplemented with skin testing and, if all tests are negative, provocation tests are performed if the child can cooperate. Each substance tested is individually evaluated, and a positive result is evaluated in relation to the timing of administration during the reaction. A final conclusion is made when all test results are available. In cases where the planned investigations are not completed, an expert assessment and guidance for re‐administration of anesthesia are provided.

### 2.3 Statistical methods

Fisher's exact test was conducted using R studio (version 4.3.1) to test differences between the test positive group and the test negative group. Tryptase values were logarithmically transformed to ensure normally distributed data, followed by Welch *t*‐tests. *p*‐values below .05 were considered statistically significant. Sensitivities and specificities were calculated using Microsoft Excel (version 16.81).

## 3 RESULTS

### 3.1 Population

The median age of the 70 included children was 14 years (range 1–17 years), and 36% were female. 97.1% of the patients were ASA class 1–2. 21.4% had atopy, and 6% had previously reported a drug allergy at the time of the reaction. No statistically significant differences in either atopy nor known drug allergies were seen when comparing test positive with test negative groups.

### 3.2 Clinical manifestations and treatment

The most common symptoms were skin symptoms (94%) followed by cardiovascular symptoms (36% had tachycardia, 33% had hypotension). When comparing the test positive group with the test negative group, there were significantly more children with hypotension and tachycardia in the test positive group (see Table 1).

**TABLE 1:** Demographic characteristics of the included patients when divided into a test positive and test negative group, and clinical manifestations of the suspected perioperative allergic reaction.

Variable	Total (*n* = 70)	Positive (*n* = 17)	Negative (*n* = 53)^a^	*p*‐Value
Age median (IQR)	14.0 (5)	15 (2)	14.0 (6)	.27
Gender				
Female (%)	25 (36)	9 (52.9)	16 (30.2)	.14
Male (%)	45 (64)	8 (47.1)	37 (69.2)	.14
ASA score				
Class 1–2 (%)	68 (97.1)	17 (100)	51 (96.2)	1.0
Class 3–4 (%)	2 (2.9)	0	2 (3.8)	1.0
Atopy (%)	15 (21.4)	4 (23.5)	11 (20.8)	1.0
Known drug allergies (%)	4 (6)	0	4 (7.5)	.57
Symptoms				
Respiratory (%)	12 (17)	4 (23.5)	8 (15.1)	.47
Hypotension (%)	23 (33)	12 (70.6)	11 (20.8)	**<.001**
Tachycardia (%)	25 (36)	10 (58.9)	15 (28.3)	.**04**
Bradycardia (%)	3 (4)	1 (5.9)	2 (3.8)	1.0
Skin (%)	66 (94)	15 (88.2)	51 (96.2)	.25
Ring & Messmer				
Grade I (%)	37 (53)	4 (23.5)	33 (62.3)	.**01**
Grade II (%)	13 (18.5)	1 (6.0)	12 (22.6)	.16
Grade III (%)	20 (28.5)	12 (70.5)	8 (15.1)	**<.0001**
Tryptase baseline median (IQR)	3.58 (1.71)	4.50 (4.3)	3.58 (1.62)	.26
Tryptase reaction median (IQR)	4.24 (6.05)	9.30 (13.18)	3.66 (3.92)	.**04**
Tryptase elevated (%)^b^	16 (22.9)	6 (60)	10 (30)	.14
Treatment				
None (%)	5 (7)	0^c^	5 (9.4)	.58
Adrenaline/noradrenaline (%)	21 (30)	10 (62.5)	11 (20.8)	**<.01**
Adrenaline and/or ephedrine (%)	24 (34.8)	11 (68.8)	13 (24.5)	.**02**
Ephedrine (%)	17 (25)	10 (62.5)	7 (13.2)	**<.001**
Antihistamines (%)	59 (85.5)	14 (87.5)	45 (84.9)	1.0
Corticosteroids (%)	43 (62)	10 (62.5)	33 (62.3)	1.0
I.V. fluids (%)	18 (26)	9 (56.3)	9 (17.0)	**<.01**

*Note*: Ring & Messmer: grade I: skin/mucosal symptoms, grade II: non‐life threatening symptoms from several organ systems such as skin, cardiovascular, respiratory, or gastrointestinal symptoms, grade III: life threatening symptoms from multiple organ systems and grade IV: circulatory/respiratory arrest. Respiratory symptoms included desaturation, stridor, and bronchospasms. Skin symptoms included urticaria, angioedema, flushing and unclassified rash.

Abbreviations: ASA, American society of Anesthesiologists; IQR, interquartile range.

^a^ 8 children in the “test negative” category did not undergo complete allergy assessment.

^b^ Elevated tryptase is defined as acute serum tryptase >1.2*basal serum tryptase+2. In total, 43 children had an acute serum tryptase taken (*n* = 10 in positive group and *n* = 33 in negative group). See Section 2 for further explanation on tryptase cut‐off.

^c^ Information on treatment was unavailable in 1 patient in the test positive group.

85.5% of all patients were treated with antihistamines, and 62% received corticosteroids, making these the most administered treatments. 30% received adrenaline/noradrenaline, and 26% received I.V. fluids. Adrenaline/noradrenaline, intravenous fluids, and ephedrine were administered statistically significantly more often in the test positive group than the test negative group.

**FIGURE 1:**
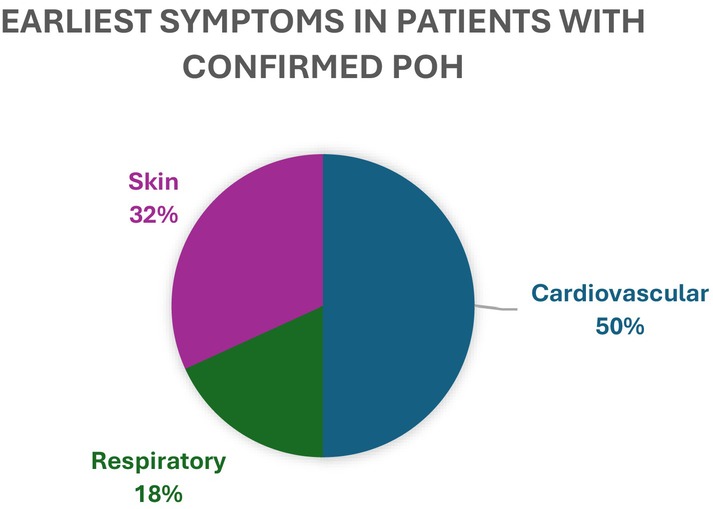
Earliest symptoms in patients with confirmed POH.

In Figure 1, a visual representation of the earliest observed symptoms in the test positive group is presented. 50% of the confirmed positive patients presented with cardiovascular symptoms (CVS), with hypotension being the most common initial symptom.

In total, 43 children (61%) had acute tryptase available. Median acute tryptase was significantly higher in the test‐positive group (*p* = .04).

Most children had a mild reaction, with 53% having a grade I, 18.5% having a grade II, and 28.5% having a grade III reaction. No patients experienced a grade IV reaction. When comparing the test positive and test negative groups, grade I reactions were statistically significantly more frequent in the test negative group (*p* = .01) and grade III was more frequent in the test positive group (*p* < .0001); see Table 1.

### 3.3 Allergy investigations

In total 17/70 (24.3%) patients tested positive, confirming an allergic reaction. 8 children did not undergo a full diagnostic workup due to young age, but the performed tests were negative and they were included in the test negative group.

In total, 8 children tested positive on specific IgE, 7 on skin prick tests, 9 on intradermal tests, 2 on provocation tests, and 1 on the histamine release test. The two positive provocation tests were pethidine and propofol. In 7 children, the culprit allergen was identified using a combination of tests.

**FIGURE 2:**
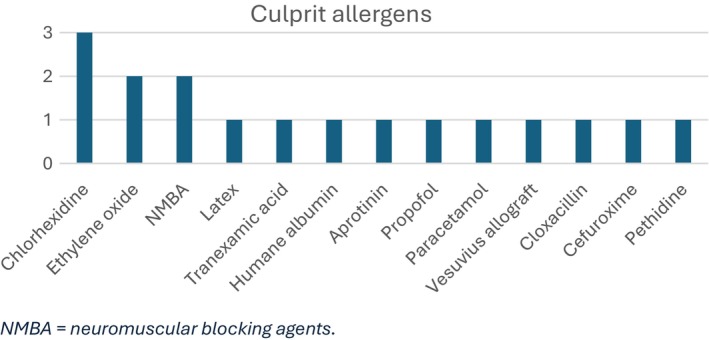
Confirmed culprit allergens. NMBA, neuromuscular blocking agents.

The most commonly confirmed allergen was chlorhexidine (*n* = 3), followed by NMBA's (*n* = 2) and antibiotics (*n* = 2), but a range of different allergens was confirmed, as shown in Figure 2.

### 3.4 Sensitivity and specificity of risk stratification algorithm for perioperative immediate hypersensitivity

Sensitivities and specificities for different variables are shown in Table 2. The risk stratification algorithm includes three criteria: elevated tryptase, two or more organ systems involved, and urticaria/angioedema, and proves to be a very sensitive predictor of perioperative hypersensitivity in our pediatric population (88.2%) but with a low specificity (17.0%). The presence of skin symptoms of any kind had the same sensitivity as the risk stratification algorithm but with a very low specificity (5.7%). High tryptase levels alone had a low sensitivity (60.0%). The combination of elevated tryptase or CVS or urticaria/angioedema had the highest sensitivity (94.1%) and a high negative predictive value (NPV) (88.9%).

**TABLE 2:** Sensitivities, specificities, positive predictive values (PPV), and negative predictive values (NPV) of different test combinations. 95% confidence intervals are in parenthesis.

Test (*n* = 70)	Sensitivity	Specificity	PPV	NPV
Elevated tryptase or 2 organ systems or U/A^a^	**88.2 (63.6–98.5)**	**17.0 (8.1–29.8)**	25.4 (21.6–29.7)	81.8 (51.8–95.0)
Elevated tryptase^b^	**60.0 (26.2–87.8)**	**69.7 (51.3–84.4)**	37.5 (22.5–55.3)	85.2 (72.3–92.7)
Involvement of 2 organ systems	76.5 (50.1–93.2)	58.5 (44.1–71.9)	37.1 (28.1–47.2)	88.6 (76.2–95.0)
U/A	58.8 (32.9–81.6)	28.3 (16.8–42.4)	20.8 (14.6–28.9)	68.2 (51.3–81.4)
SS	**88.2 (63.6–98.5)**	5.7 (1.2–15.7)	23.1 (20.0–26.5)	60.0 (21.4–89.2)
CVS	75.0 (47.6–92.7)	66.0 (51.7–78.5)	40.0 (29.4–51.6)	89.7 (78.4–95.4)
RS	23.5 (6.8–49.9)	84.5 (72.4–93.3)	33.3 (14.7–59.3)	77.6 (72.2–82.2)
Elevated tryptase + CVS^b^	50.0 (18.7–81.3)	84.9 (68.1–94.9)	50.0 (26.5–73.4)	84.9 (74.8–91.4)
Elevated tryptase + SS^b^	60.0 (26.2–87.8)	69.7 (51.3–84.4)	37.5 (22.5–55.3)	85.2 (72.3–92.7)
CVS + SS	64.7 (38.3–85.8)	67.9 (53.7–80.1)	39.3 (27.7–52.3)	85.7 (75.4–92.1)
CVS + RS	23.5 (6.8–49.9)	94.3 (84.3–98.9)	57.1 (24.9–84.3)	79.4 (74.6–83.5)
RS + SS	17.7 (3.8–43.4)	88.7 (77.0–95.7)	33.3 (12.3–64.1)	77.1 (72.5–81.0)
Elevated tryptase +2 organ systems + SS^b^	50.0 (18.7–81.3)	84.9 (68.1–94.9)	50.0 (26.5–73.4)	84.9 (74.8–91.4)
Elevated tryptase +2 organ systems^b^	50.0 (18.7–81.3)	84.9 (68.1–94.9)	50.0 (26.5–73.4)	84.9 (74.8–91.4)
Elevated tryptase + RS^b^	20.0 (2.5–55.6)	93.9 (79.8–99.3)	50.0 (13.9–86.2)	79.5 (73.7–84.2)
Elevated tryptase or RS or SS	94.1 (71.3–99.9)	1.9 (0.05–10.1)	23.5 (21.4–25.6)	50.0 (6.2–93.8)
Elevated tryptase or RS or U/A	76.5 (50.1–93.2)	20.8 (10.8–34.1)	23.6 (18.7–29.4)	73.3 (50.2–88.3)
Elevated tryptase or CVS or U/A	**94.1 (71.3–99.9)**	15.1 (6.8–27.6)	26.2 (23.2–29.5)	**88.9 (51.8–98.4)**

Abbreviations: CVS, cardiovascular symptoms (any); RS, respiratory symptoms (any); SS, skin symptoms (any); U/A, urticaria and/or angioedema.

^a^ Risk stratification algorithml.^14^

^b^ *N* = 43 due to limited data on tryptase.

## 4 DISCUSSION

### 4.1 Main findings

In this retrospective single center study, we have investigated POH in children using data from the highly specialized national referral center in Denmark, and 17/70 (24.3%) of the children tested positive.

94% of the children experienced skin symptoms. Skin symptoms might be difficult to acknowledge in the operating theater as most of the body is usually covered by surgical drapes. In addition, the type of rash, for example, urticaria, may be difficult to identify. No significant difference was seen between test positive and test negative groups in skin symptoms, which may be because clinically allergic and non‐allergic skin symptoms can be difficult to distinguish, and therefore patients are referred for allergy testing. Cardiovascular symptoms such as hypotension or tachycardia are often seen during anesthesia related to anesthetic and/or surgical management. When hypotension and tachycardia occur unexpectedly and together, this can be a sign of POH.^3^, ^16^ In the test positive group, more children experienced both hypotension and tachycardia when compared to the test negative group. Furthermore, cardiovascular symptoms were the initial symptoms in 50% of the children that tested positive, followed by skin symptoms (32%) and respiratory symptoms (18%). In a retrospective multicenter cohort study^9^ including 29 children, cardiovascular symptoms were also the first symptoms; however, here, skin symptoms were less common. Similarly to this, the NAP6 study,^4^ which included 11 children, also showed hypotension as the dominant symptom in children, but with respiratory symptoms appearing early. However, both studies only included grade III and IV reactions; however, in our study, we include all reaction grades, and interestingly, only 2.9% had grade III‐IV reactions. In a Turkish retrospective single center cohort study including 50 children,^10^ the most common symptoms were skin symptoms. The same pattern was seen in a prospective single center cohort study including 29 children.^5^ Both Turkish studies featured all reaction grades and are thus more comparable to our study in symptomology.

In our study, 53% of the patients experienced a grade I reaction. Significantly more children in the test negative group experienced a grade I reaction, and more children in the test positive group experienced a grade III reaction. This indicates that although confirmed POH in children is uncommon, it may be more severe, further emphasizing the need for quick recognition and treatment.

In our study, measurement of tryptase in the acute phase was included in 43/70 (61%) of cases. Tryptase is a sensitive indicator for mast cell activation when taken 30 min–2 h after symptom onset,^16^, ^17^ and it is therefore important that clinicians consider the test as soon as the patient is stable. In a retrospective single‐center study from Singapore including 15 children,^12^ tryptase was taken in 53% of the children, and in the retrospective multicenter cohort study^9^ it was taken in 72% of the children, which implies that there is a need for increased education of anesthesiologists to ensure serum tryptase samples are taken correctly.

The test positive group in this study had a significantly higher median acute tryptase level when compared with the test negative group. Elevated tryptase was seen in 10 children who tested negative; two did not undergo a complete testing program, and a culprit drug could have been missed. Other reasons for elevated tryptase with negative testing could be due to false negative testing, either due to incomplete reporting of the allergens the patient was exposed to or perhaps due to low sensitivity of the allergy tests. On the other hand, 4 children tested positive despite not having a rise in acute tryptase, which could be because their reactions were so mild that it was not reflected in their tryptase. Another explanation could be false positive testing, which may occur if allergy test results are being overinterpreted by clinicians, or if skin test concentrations are too high and irritant to the skin. To ensure the most accurate diagnosis, drug provocation should, as we aim to, be performed in cases of inconclusive testing. However, drug provocation is not routinely performed in pediatric patients in most perioperative allergy testing centers.

All calculations on elevated tryptase in this study are based on the adult consensus formula (acute serum tryptase >1.2*basal serum tryptase+2)^16^ but a new consensus formula on tryptase specifically for children (acute serum tryptase>basal serum tryptase+0.71) has recently been proposed^18^ and may be more appropriate. 48 of the children from our pediatric population were included in that study, and therefore this new formula cannot be reliably tested on our population.

Overall, antihistamines were the most commonly administered medication in both groups, followed by corticosteroids. It is recommended that all patients with grade II‐IV reactions be administered diluted I.V. adrenaline and I.V. fluids, and antihistamines and corticosteroids are the second line of treatment for these reactions.^1^ Most of the test‐positive group experienced a grade II or III reaction (76.5%), only 62.5% received adrenaline and 56.3% received I.V. fluids. A substantial number of children in all groups received ephedrine, and although not anaphylaxis‐specific treatment, it is the first line of treatment for hypotension during anesthesia in Denmark. Since POH is such a rare occurrence, and hypotension occurs commonly during anesthesia, it makes sense for anesthesiologists to use ephedrine or other vasopressors first and reserve adrenaline for lack of response to other vasopressors or for cases of overt anaphylaxis. This approach was also suggested in the NAP6 study.^4^

A total of 12 different culprit allergens were confirmed, with chlorhexidine being the most common (*n* = 3). Two children tested positive to NMBA's, antibiotics and ethylene oxide, respectively. This differs from other published studies, where NMBA's^9^, ^10^, ^11^ or antibiotics^4^, ^12^, ^13^ were the most common culprit allergens. Chlorhexidine is routinely and widely used as a disinfectant in Danish health care settings, including operating theaters, and consequently, it is one of the most common culprit allergens in Denmark.^19^ Ethylene oxide is often used as a sterilizing agent. Exposure to this is usually not documented^20^ and therefore all patients investigated in DAAC are tested with this. Furthermore, some uncommon allergens were identified, such as paracetamol and propofol, which further emphasizes the importance of identifying and testing all suspected drugs and substances. One child tested positive for latex allergy. In previous studies, latex was often highlighted as one of the most common allergens for POH, but newer studies seem to identify few to no latex allergy sufferers.^21^ Reasons for this could be a greater awareness of latex allergy among both healthcare workers and manufacturers of medical supplies, which has reduced allergen content as well as provided many latex‐free and powder‐free utensils.^2^

### 4.2 Use of the risk stratification algorithm in pediatric patients

High sensitivity is a key element in an algorithm made for predicting a positive diagnosis of POH and allergy in general, as it is important to identify as many of the true positive patients as possible. If sensitivity is low and the patient scores negative on the risk stratification algorithm, but it turns out to be a false negative result, the patient will not be referred for allergy investigation and might be exposed to the allergen again and may experience another harmful allergic reaction. On the other hand, a false positive result on the risk stratification evaluation will lead to referral for allergy testing, and this then poses a risk of false positive testing, which would mean restrictions on which drugs can be used for subsequent exposure to anesthesia.

The risk stratification algorithm, which includes elevated tryptase or involvement of two or more organ systems or urticaria/angioedema,^14^ along with several other possible combinations for POH has been presented based on the children from the DAAC database. In a similar study^15^ where both adults and children were included, a sensitivity of 98.8% was found, which was the highest sensitivity of their test combinations, and a specificity of 34.6% for the risk stratification algorithm. Our study also showed a high sensitivity of 88.2% for the risk stratification algorithm, and a low specificity of 17%. This high sensitivity indicates the algorithm's strong ability to correctly identify individuals at risk, though it may lead to a higher number of false positives due to the trade‐off with specificity. “Any skin symptoms” had the same sensitivity but with a lower specificity because skin symptoms were very frequent symptoms among all the referred patients. A higher sensitivity of 94.1% was seen when combining “elevated tryptase or cardiovascular symptoms or urticaria/angioedema”. This combination had almost the same specificity and a higher NPV than the risk stratification algorithm. Due to the small population size, it is difficult to say whether this is a coincidence or if it this may be a more appropriate combination for pediatric patients. It is important to note that an algorithm for POH cannot independently determine if a reaction is POH or if a patient should be referred for allergy workup. The role of an algorithm is to assist clinicians, but referrals should always rely on individual patient assessment.

### 4.3 Study strengths and limitations

Here, a large single‐center study is presented, and all children underwent systematic investigation in a national reference center. Testing was done interdisciplinary, as the team at DAAC consists of both allergologists and anesthesiologists, which may make a correct assessment and diagnosis more likely. Furthermore, this study includes all reaction grades, which may provide a more accurate representation of POH in children.

Another advantage is that drug provocation tests were done when skin testing was inconclusive or negative and there was a need for further testing, which provides a safer diagnosis. To our knowledge, aside from this study, provocation in pediatric patients is only done in a few studies.^5^, ^10^

The retrospective design is a limitation, since it might cause uncertainties as data has not been collected specifically for this study. Even if higher than in other similar studies, the size of the population makes it difficult to draw firm conclusions, which could improve the diagnostic algorithm. Another limitation is that 8 children did not complete full investigations due to young age. All 8 had SPT, 3 did not have ICT, and none of the 8 had provocation tests performed. The possibility of POH cannot fully be excluded in these cases as they did not complete testing.

## 5 CONCLUSION

In conclusion, POH in children is rare and in this Danish cohort they only accounted for 8.3% of patients referred for perioperative allergy investigations. In addition, only 24.3% of the children tested positive, but they had significantly more serious (grade III) reactions, with hypotension often appearing as the first symptom. This emphasizes the need for anesthesiologists to be aware of anaphylaxis as a differential diagnosis to perioperative hypotension.

Due to the sample size, it was not possible to reliably identify risk factors for POH in children, and this should be the focus of future studies.

## AUTHOR CONTRIBUTIONS

**Cecilie N. Madsen:** Conceptualization; data curation; formal analysis; writing – original draft; writing – review and editing; methodology. **Birgitte Bech Melchiors:** Writing – review and editing; data curation. **Holger Mosbech:** Writing – review and editing; data curation. **Kirsten Skamstrup Hansen:** Writing – review and editing. **Lene H. Garvey:** Conceptualization; data curation; writing – review and editing; supervision; methodology.

## FUNDING INFORMATION

This study received no external funding.

## CONFLICT OF INTEREST STATEMENT

None of the authors have any conflict of interests in relation to this manuscript.

### PEER REVIEW

The peer review history for this article is available at https://www.webofscience.com/api/gateway/wos/peer‐review/10.1111/pai.70079.
